# Supplementary material for: Leaf functional traits and pathogens: Linking coffee leaf rust with intraspecific trait variation in diversified agroecosystems
Source: PLoS One. 2023 Apr 13;18(4):e0284203. doi: 10.1371/journal.pone.0284203 (PMC10101423; doi:10.1371/journal.pone.0284203)
Supplement: S1 Table — (PDF) [file pone.0284203.s002.pdf]

S2 Table. Bivariate standardized major axis regression analyses among coffee leaf rust severity (CLR) and leaf traits in *Coffea arabica* (T = leaf thickness; S = stomatal density; LA = leaf area; SLA = specific leaf area; LDMC = leaf dry matter content; LN = leaf nitrogen concentration; CN = leaf carbon to nitrogen ratio), across all shade tree treatments in the (a) top stratum (>70% of total plant height), and (b) bottom stratum (<40% of total plant height). Model r^2 and P-values (in brackets) for each bivariate model are presented in the lower left section of the matrix, with significant relationships ($P < 0.05$) highlighted in bold. The upper right section of the matrix presents model slopes and associated 95% confidence intervals (in brackets).

(a)	Disease metric	Morphological leaf traits					Chemical leaf traits	
	log CLR	log T	S	LA	log SLA	log LDMC	LN	log CN
log CLR	--	4.67 (4.29, 5.09)	-0.01 (-0.01, -0.02)	-1.30 (-1.41, -1.19)	0.14 (0.13, 0.16)	-0.01 (-0.013, -0.011)	-1.42 (-1.54, -1.30)	4.04 (3.71, 4.40)
log T	0.000 (0.941)	--	0.003 (0.003, 0.004)	0.28 (0.26, 0.30)	-0.03 (-0.03, -0.03)	0.002 (0.002, 0.003)	0.30 (0.28, 0.33)	-0.87 (-0.94, -0.80)
S	0.006 (0.327)	0.004 (0.402)	--	-92.84 (-107.03, -80.53)	-11.33 (-13.07, -9.82)	0.83 (0.72, 0.95)	-96.49 (-111.65, -83.39)	275.62 (238.57, 318.43)
LA	**0.031 (<0.001)**	**0.065 (<0.001)**	**0.106 (<0.001)**	--	0.11 (0.10, 0.12)	-0.01 (-0.01, -0.01)	1.09 (1.00, 1.18)	-3.11 (-3.37, -2.87)
log SLA	0.000 (0.829)	**0.082 (<0.001)**	**0.094 (<0.001)**	**0.043 (<0.001)**	--	-0.08 (-0.08, -0.07)	9.89 (9.11, 10.73)	-28.24 (-30.64, -26.02)
log LDMC	0.002 (0.939)	**0.013 (0.012)**	**0.152 (<0.001)**	**0.037 (<0.001)**	**0.511 (<0.001)**	--	-123.75 (-133.87, -114.4)	353.42 (326.80, 382.22)
LN	**0.044 (<0.001)**	0.003 (0.185)	**0.058 (0.001)**	**0.101 (<0.001)**	**0.114 (<0.001)**	**0.175 (<0.001)**	--	-2.86 (-2.89, -2.82)
log CN	**0.037 (<0.001)**	**0.007 (0.064)**	**0.079 (0.002)**	**0.120 (<0.001)**	**0.106 (<0.001)**	**0.182 (<0.001)**	**0.981 (<0.001)**	--

(b)	Disease metric	Morphological leaf traits					Chemical leaf traits	
	log CLR	log T	S	log LA	log SLA	LDMC	LN	log CN
log CLR	--	-6.08<br>(-6.63, -5.58)	-0.019<br>(-0.022, -0.016)	-1.74<br>(-1.89, -1.61)	-3.38<br>(-3.69, -3.11)	0.02<br>(0.01, 0.02)	-1.63<br>(-1.77, -1.51)	4.81<br>(4.43, 5.22)
log T	0.004<br>(0.155)	--	0.003<br>(0.003, 0.004)	0.29<br>(0.26, 0.31)	-0.56<br>(-0.61, -0.51)	-0.002<br>(-0.003, -0.002)	0.27<br>(0.25, 0.29)	-0.79<br>(-0.86, -0.73)
S	0.000<br>(0.797)	0.000<br>(0.960)	--	-82.42<br>(-95.76, -70.94)	-154.70<br>(-179.36, -133.42)	0.72<br>(0.62, 0.84)	-72.90<br>(-84.55, -62.84)	214.59<br>(185.09, 248.79)
log LA	**0.134**<br>**(<0.001)**	**0.099**<br>**(<0.001)**	0.005<br>(0.346)	--	1.95<br>(1.79, 2.12)	-0.01<br>(-0.01, -0.01)	0.94<br>(0.86, 1.02)	-2.76<br>(-3.01, -2.54)
log SLA	**0.019**<br>**(0.002)**	**0.024**<br>**(<0.001)**	**0.032**<br>**(0.018)**	**0.023**<br>**(<0.001)**	--	-0.005<br>(-0.005, -0.004)	0.48<br>(0.44, 0.52)	-1.42<br>(-1.54, -1.31)
LDMC	**0.011**<br>**(0.02)**	**0.016**<br>**(0.004)**	0.019<br>(0.069)	**0.018**<br>**(0.002)**	**0.325**<br>**(<0.001)**	--	-107.81<br>(-116.84, -99.48)	317.02<br>(292.39, 343.72)
LN	**0.103**<br>**(<0.001)**	0.008<br>(0.045)	0.027<br>(0.031)	**0.014**<br>**(0.006)**	**0.076**<br>**(<0.001)**	**0.133**<br>**(<0.001)**	--	-2.94<br>(-2.98, -2.90)
log CN	**0.095**<br>**(<0.001)**	0.008<br>(0.036)	0.033<br>(0.016)	**0.021**<br>**(0.001)**	**0.075**<br>**(<0.001)**	**0.124**<br>**(<0.001)**	**0.975**<br>**(<0.001)**	--
