# Supplementary material for: Leaf functional traits and pathogens: Linking coffee leaf rust with intraspecific trait variation in diversified agroecosystems
Source: PLoS One. 2023 Apr 13;18(4):e0284203. doi: 10.1371/journal.pone.0284203 (PMC10101423; doi:10.1371/journal.pone.0284203)

PLOS ONE

Leaf functional traits and pathogens: how foliar disease shapes intraspecific trait variation in agroecosystems

--Manuscript Draft--

Manuscript Number:	PONE-D-22-31465
Article Type:	Research Article
Full Title:	Leaf functional traits and pathogens: how foliar disease shapes intraspecific trait variation in agroecosystems
Short Title:	Leaf functional traits and pathogens
Corresponding Author:	Marney E. Isaac<br>University of Toronto<br>Toronto, Ontario CANADA
Keywords:	Agroforestry; Coffea arabica; coffee; coffee leaf rust; foliar biotrophic fungi; fungal disease severity; global functional trait databases; Hemileia vastatrix; resource acquisition strategies
Abstract:	Research has demonstrated that intraspecific functional trait variation underpins plant responses to environmental variability. However, remarkably few studies have evaluated how trait variation shifts in response to plant pathogens, despite evidence of plants expressing distinct plant strategies in response to pathogen pressures. Understanding trait-pathogen relationships are critical to the success of economically significant agricultural systems and can further inform global patterns of functional trait variation. We examined leaf intraspecific trait variability (ITV) in response to foliar disease severity, using Coffea arabica as a model species. We quantified coffee leaf rust (CLR) severity—a fungal disease prominent in coffee systems—and measured key coffee leaf functional traits under contrasting, but widespread, management conditions in a biodiverse agroecosystem. We found that coffee plants express significant ITV, which is largely related to shade tree treatment and leaf position within coffee canopy strata. Yet within a single plant canopy stratum, CLR severity increased with increasing resource conserving trait values. However, coffee leaves in diseased environments expressed overall greater resource acquiring trait values, as compared to plants in non-diseased systems. We provide among the first evidence demonstrating that leaf traits are correlated with foliar disease severity in crops, and that functional trait relationships and syndromes shift in response to increased disease prevalence in plants. In doing so, we address a vital gap in our collective understanding of global patterns of functional trait variation and highlight the need to further explore the potential role of pathogens in codifying or destabilizing established global trait relationships and spectra.
Order of Authors:	Stephanie Gagliardi
	Jacques Avelino
	Adam Martin
	Marc Cadotte
	Elias de Melo Virginio Filho
	Marney Isaac
Additional Information:	
**Question**	**Response**
**Financial Disclosure**<br><br>Enter a financial disclosure statement that describes the sources of funding for the work included in this submission. Review the submission guidelines for detailed	This work was supported by the Natural Sciences and Engineering Research Council of Canada (Discovery Grant to M.E.I. and Alexander Graham Bell Canada Graduate Scholarship to S.G.). The funders had no role in study design, data collection and analysis, decision to publish, or preparation of the manuscript.

Powered by Editorial Manager® and ProduXion Manager® from Aries Systems Corporation

requirements. View published research articles from *PLOS ONE* for specific examples.<br><br>This statement is required for submission and **will appear in the published article** if the submission is accepted. Please make sure it is accurate.<br><br>**Unfunded studies**<br>Enter: *The author(s) received no specific funding for this work.*<br><br>**Funded studies**<br>Enter a statement with the following details:<br>• Initials of the authors who received each award<br>• Grant numbers awarded to each author<br>• The full name of each funder<br>• URL of each funder website<br>• Did the sponsors or funders play any role in the study design, data collection and analysis, decision to publish, or preparation of the manuscript?<br>• **NO** - Include this sentence at the end of your statement: *The funders had no role in study design, data collection and analysis, decision to publish, or preparation of the manuscript.*<br>• **YES** - Specify the role(s) played.<br><br>* typeset	
**Competing Interests**<br><br>Use the instructions below to enter a competing interest statement for this submission. On behalf of all authors, disclose any competing interests that could be perceived to bias this work—acknowledging all financial support and any other relevant financial or non-financial competing interests.<br><br>This statement is required for submission and **will appear in the published article** if the submission is accepted. Please make sure it is accurate and that any funding sources listed in your Funding Information later in the submission form are also declared in your Financial Disclosure	The authors have declared that no competing interests exist.

statement.<br><br>View published research articles from *PLOS ONE* for specific examples.<br><br>**NO authors have competing interests**<br><br>Enter: *The authors have declared that no competing interests exist.*<br><br>**Authors with competing interests**<br><br>Enter competing interest details beginning with this statement:<br><br>*I have read the journal's policy and the authors of this manuscript have the following competing interests: [insert competing interests here]*<br><br>* typeset	
**Ethics Statement**<br><br>Enter an ethics statement for this submission. This statement is required if the study involved:<br><br>• Human participants<br>• Human specimens or tissue<br>• Vertebrate animals or cephalopods<br>• Vertebrate embryos or tissues<br>• Field research<br><br>Write "N/A" if the submission does not require an ethics statement.<br><br>General guidance is provided below. Consult the submission guidelines for detailed instructions. **Make sure that all information entered here is included in the Methods section of the manuscript.**	n/a

**Format for specific study types**<br><br>**Human Subject Research (involving human participants and/or tissue)**<br>• Give the name of the institutional review board or ethics committee that approved the study<br>• Include the approval number and/or a statement indicating approval of this research<br>• Indicate the form of consent obtained (written/oral) or the reason that consent was not obtained (e.g. the data were analyzed anonymously)<br><br>**Animal Research (involving vertebrate animals, embryos or tissues)**<br>• Provide the name of the Institutional Animal Care and Use Committee (IACUC) or other relevant ethics board that reviewed the study protocol, and indicate whether they approved this research or granted a formal waiver of ethical approval<br>• Include an approval number if one was obtained<br>• If the study involved *non-human primates*, add *additional details* about animal welfare and steps taken to ameliorate suffering<br>• If anesthesia, euthanasia, or any kind of animal sacrifice is part of the study, include briefly which substances and/or methods were applied<br><br>**Field Research**<br><br>Include the following details if this study involves the collection of plant, animal, or other materials from a natural setting:<br>• Field permit number<br>• Name of the institution or relevant body that granted permission	
**Data Availability**<br><br>Authors are required to make all data underlying the findings described fully available, without restriction, and from the time of publication. PLOS allows rare exceptions to address legal and ethical concerns. See the PLOS Data Policy and FAQ for detailed information.	Yes - all data are fully available without restriction

A Data Availability Statement describing where the data can be found is required at submission. Your answers to this question constitute the Data Availability Statement and **will be published in the article**, if accepted.<br><br>**Important:** Stating 'data available on request from the author' is not sufficient. If your data are only available upon request, select 'No' for the first question and explain your exceptional situation in the text box.<br><br>Do the authors confirm that all data underlying the findings described in their manuscript are fully available without restriction?	
**Describe where the data may be found in full sentences. If you are copying our sample text, replace any instances of XXX with the appropriate details.**<br><br>• If the data are **held or will be held in a public repository**, include URLs, accession numbers or DOIs. If this information will only be available after acceptance, indicate this by ticking the box below. For example: *All XXX files are available from the XXX database (accession number(s) XXX, XXX.*).<br>• If the data are all contained **within the manuscript and/or Supporting Information files**, enter the following: *All relevant data are within the manuscript and its Supporting Information files.*<br>• If neither of these applies but you are able to provide **details of access elsewhere**, with or without limitations, please do so. For example:<br><br>*Data cannot be shared publicly because of [XXX]. Data are available from the XXX Institutional Data Access / Ethics Committee (contact via XXX) for researchers who meet the criteria for access to confidential data.*<br><br>*The data underlying the results presented in the study are available from (include the name of the third party*	The data underlying the results presented in the study are available from the TRY functional trait database at try-db.org.

and contact information or URL).<br>• This text is appropriate if the data are owned by a third party and authors do not have permission to share the data.<br><br>* typeset	
Additional data availability information:	Tick here if the URLs/accession numbers/DOIs will be available only after acceptance of the manuscript for publication so that we can ensure their inclusion before publication.

Powered by Editorial Manager® and ProduXion Manager® from Aries Systems Corporation

Manuscript

Click here to access/download;Manuscript;Gagliardi et al. PLOS ONE - manuscript file.docx

Leaf functional traits and pathogens: How foliar disease shapes intraspecific trait variation in agroecosystems

Stephanie Gagliardi[1], Jacques Avelino[2, 3, 4], Adam R Martin[1], Marc Cadotte[1], Elias de Melo Virginio Filho[4], Marney E Isaac[1*]

[1] Department of Physical and Environmental Sciences, University of Toronto Scarborough, Toronto, Ontario, Canada

[2] CIRAD, UMR PHIM, Turrialba, Costa Rica

[3] PRISM, Plant Health Institute of Montpellier, Montpellier, France

[4] Centro Agronómico Tropical de Investigación y Enseñanza, Turrialba, Costa Rica

*Corresponding author

E-mail: marney.isaac@utoronto.ca (MEI)

Author Contribution

SG, JA, and MEI planned and designed the research. SG conducted fieldwork and analyzed data, with supervision by JA and MEI, and resources from JA, EMVF, and MEI. All authors contributed to the writing, review and editing process.

Abstract

Research has demonstrated that intraspecific functional trait variation underpins plant responses to environmental variability. However, remarkably few studies have evaluated how trait variation shifts in response to plant pathogens, despite evidence of plants expressing distinct plant strategies in response to pathogen pressures. Understanding trait-pathogen relationships are critical to the success of economically significant agricultural systems and can further inform global patterns of functional trait variation. We examined leaf intraspecific trait variability (ITV) in response to foliar disease severity, using *Coffea arabica* as a model species. We quantified coffee leaf rust (CLR) severity—a fungal disease prominent in coffee systems—and measured key coffee leaf functional traits under contrasting, but widespread, management conditions in a biodiverse agroecosystem. We found that coffee plants express significant ITV, which is largely related to shade tree treatment and leaf position within coffee canopy strata. Yet within a single plant canopy stratum, CLR severity increased with increasing resource conserving trait values. However, coffee leaves in diseased environments expressed overall greater resource acquiring trait values, as compared to plants in non-diseased systems. We provide among the first evidence demonstrating that leaf traits are correlated with foliar disease severity in crops, and that functional trait relationships and syndromes shift in response to increased disease prevalence in plants. In doing so, we address a vital gap in our collective understanding of global patterns of functional trait variation and highlight the need to further explore the potential role of pathogens in codifying or destabilizing established global trait relationships and spectra.

Keywords: Agroforestry, *Coffea arabica*, coffee, coffee leaf rust, foliar biotrophic fungi, fungal disease severity, global functional trait databases, *Hemileia vastatrix*, resource acquisition strategies

Introduction

Over the last few decades, there have been a growing number of studies quantifying and describing plant functional trait relationships among and within plant species, e.g. [1,2]. Trait relationships and trait spectra, such as those comprising the “Leaf Economics Spectrum” (LES) [1], are now commonly interpreted to reflect differences in plant resource-use strategies, such that leaves and plant species differ from one another across a gradient of resource conservation and resource acquisition. Drivers of trait variation include abiotic conditions, biotic interactions, and anthropogenic influences, all of which individually or cumulatively provide insights into the determinants of species distributions [3,4], and enhance our understanding of the role plant diversity plays in governing rates of ecosystem functioning [1,5,6].

However, research on functional trait relationships—and indeed much of the world’s functional trait data—has largely focused only on quantifying traits in “healthy” plants or ecosystems [7]. In fact, standardized methodologies for measuring plant functional traits explicitly call for the exclusion of plants with visible signs of herbivore damage and pathogen incidence [8,9], creating a major gap in global plant functional trait databases. This is despite the fact that among the most prominent abiotic and biotic pressures on plant communities associated with global environmental change, are plant pests and pathogens [10].

Across plant species, key leaf functional traits have been established as proxies for anti-herbivore defensive strategies, including decreased specific leaf area (SLA) and increased leaf

dry matter content (LDMC), which negatively impact the palatability of leaves [11]. However, correlations between leaf functional traits and plant pathogens have not been as well established, despite emerging research linking the two. For example, within a single plant species, leaves that express greater SLA are more susceptible to, and less tolerant of, foliar biotrophic fungi (i.e., a type of pathogenic fungus that requires living host tissues to survive) [12,13]. Similarly, intraspecific variability in leaf nutrient concentrations can dictate fungal foliar disease severity [14], such that leaves with greater leaf nitrogen concentrations (LN) are associated with greater foliar biotrophic fungal disease severity [15].

Although these preliminary findings are specific to plant genotypes [16] and pathogen types [17], there is evidence indicating that leaf functional trait syndromes can shift in relation to foliar pathogens: more resource acquisitive traits relate to greater susceptibility to, and lower tolerance of, biotrophic fungal pathogens. By extension, therefore, we may also expect a shift in functional trait relationships towards acquisitive traits in leaves with visible signs of foliar disease, compared to visibly “healthy” leaves. However, since diseased leaves among and between species have been purposely excluded from many of the most prominent analyses on global leaf trait spectra [5], trait-based responses to or interactions with pathogens remain poorly assessed. As a result, we have a vital gap in our current global plant functional trait databases, and an incomplete understanding of how foliar diseases interact with leaf trait expression and their potential to codify or destabilize established global leaf trait relationships and spectra.

Our study aims to quantify intraspecific trait variation (ITV) in leaves with visible signs of foliar biotrophic fungal pathogen incidence and determine if leaf traits and trait trade-offs are correlated with foliar disease across multiple scales. To do so, we measure seven key leaf traits of a single plant cultivar, across three plant canopy strata, over a nested sampling design

comprised of multiple management practices (including shade tree diversity, amendment treatments and yield classes); a sampling scheme that was designed to maximize the extent of leaf trait variability. We use bivariate and multivariate analyses, as well as a novel application of hierarchical clustering analyses to infer relationships between pathogens and ITV. Finally, we compare trait relationships measured within diseased leaves with published trait relationships measured on visibly disease-free leaves of the same genotype. We hypothesize that (1) leaves with visible signs of foliar disease differ from one another, from resource conserving through to resource acquiring trait values, among contrasting management practices and location within the plant; (2) resource conserving traits correlate with reduced foliar disease severity; and (3) trait relationships differ in plants with foliar disease, compared to leaves with no visible signs of foliar disease.

Materials and Methods

Model species

Coffea arabica cv. Caturra is an internationally significant coffee cultivar that often expresses substantial leaf ITV resulting from both biotic and abiotic factors, including light availability [18], air temperature [19], soil moisture and nutrients [20,21], microbial diversity [22], and reproduction [23]. Additionally, coffee leaf traits have been found to co-vary along an intraspecific LES, with plants showing trait values reflecting resource conservative through to acquisitive strategies [21,23–25]. However, virtually all studies on coffee ITV have explicitly excluded plants with visible signs of pests and pathogens [21]. This concern is likely true for other wild and cultivated plant species, e.g. [26].

The Caturra variety is highly susceptible to coffee leaf rust (CLR), a foliar disease caused by the biotrophic fungus *Hemileia vastatrix*, the lifecycle of which has been extensively described and reviewed [27–29] Similar to coffee leaf ITV, microclimate conditions, which are shaped by agroecosystem design [30,31], largely constrain different lifecycle stages of *H. vastatrix* [32]. Generally, *H. vastatrix* uredospores, the only known infectious unit of *H. vastatrix*, germinate on the underside of coffee leaves and penetrate the leaf via stomata given appropriate temperatures, radiation, and moisture levels. After colonizing and maturing within the inter- and intracellular leaf space, fungal sori emerge from the stomata and produce new spores that are dispersed largely via wind, rain, and the movement of farm workers.

The essential role of coffee leaf stomata in the infection cycle suggests that greater stomatal density and improved water status of leaves increase *H. vastatrix* incidence [28]. Simultaneously, increased stomatal density is allometrically related to other leaf traits, including lower SLA and greater lamina thickness [33]. There is evidence of biochemical responses to *H. vastatrix* invasion within coffee leaves, including an increased production of defence-related proteins and hypersensitive cell death, though these responses generally occur too late in genetically susceptible coffee varieties, such as Caturra, to prevent negative consequences of CLR infection [34]. Overall, previous studies have found that high-yielding coffee plants are more susceptible to CLR [35], potentially due to the reallocation of defensive phenolic compounds from leaves to fruits [36]. However, our understanding of the role CLR plays in determining functional trait expression and trait-trait relationships is lacking, despite the role trait expression may play in moderating plant-pathogen dynamics.

Site description and experimental design

This study was conducted at the international coffee agroforestry research site Centro Agronómico Tropical de Investigación y Enseñanza (CATIE), located in Turrialba, Costa Rica (09°53´44” N, 83°40´7” W), situated at 685 m a.s.l. Sampling occurred in July 2017 during the rainy season in this region, which averages 3200 mm of annual rainfall [37]. The CATIE site is organized into three distinct blocks, with different soil amendments and shade tree treatments repeated within each of these blocks.

At CATIE we employed a nested experimental design, in efforts to quantify the full extent of potential ITV of coffee leaves, thereby enhancing our understanding of ITV across different levels of biological or spatial organization [38]. We sampled coffee plants from each of the three blocks at the CATIE site. Within each block, we sampled plants growing in two amendment regimes: moderate conventional (with applications of chemical soil fertilizer, foliar minerals, and foliar copper-based fungicide with cyproconazole), and intensive organic management (with applications of chicken manure soil fertilizer and foliar Bordeaux mixture fungicide).

Within each amendment regime, we sampled from five different shade tree treatments: coffee shaded under single-species canopies of *Erythrina poeppigiana* (Legume 1), *Chloroleucon eurycyclum* (Legume 2), and *Terminalia amazonia* (Timber) and shaded under double-species canopies of *E. poeppigiana* + *C. eurycyclum* (Legume 1 + Legume 2) and *E. poeppigiana* + *T. amazonia* (Legume 1 + Timber) (Fig 1a). Shade trees included in this study included both timber and leguminous species, a common practice in coffee agroforestry systems [39].

Fig 1. (**a**) Research design configurations with *Erythrina poeppigiana* (Legume 1), *Terminalia amazonia* (Timber), *Chloroleucon eurycyclum* (Legume 2), *E. poeppigiana* + *T. amazonia*

(Legume 1 + Timber) and *E. poeppigiana* + *C. eurycyclum* (Legume 1 + Legume 2). Dotted boxes represent study plot boundaries for all measurements. (**b**) Coffee plant profile with each of the plant strata highlighted (top, middle, bottom).

Within each shade tree treatment, we sampled coffee plants that fell into one of two yield classes: a low fruiting class (0-200 fruiting nodes), and a high fruiting class (200+ fruiting nodes), as determined by counting fruiting nodes [40], and based on a similar threshold employed by [35]. Within each yield class, three coffee plants were selected of similar age since stump-pruning (between 3-4 years old), as estimated by stem diameter [41]. All coffee plants were within a representative area of ≤10 m diameter, either directly beneath the canopy of a representative shade tree in single-species canopies, or beneath the overlapping canopies of representative shade trees in double-species canopies (Fig 1a). To capture potential intra-plant variability [42], we sampled leaves from different branches within each coffee plant from top (>70% of total plant height), middle (40-70% of total plant height), and bottom (<40% of total plant height) canopy strata (Fig 1b). Within each stratum, we then selected the first three recently developed, fully expanded coffee leaves from the branch tip that demonstrated signs of CLR infection for leaf trait measurements.

Coffee leaf measurements

We measured seven morphological and chemical leaf functional traits for each sampled coffee leaf, following protocols of [8]: leaf area (cm^2) using ImageJ software (1.47 bundled with 64-bit Java), specific leaf area (mg mm^{-2}), leaf dry matter content (LDMC; mg g^{-1}), and leaf thickness (mm), averaged from measurements collected near the tip, middle and base of the leaf using

electronic calipers. Stomatal density (number of stomates mm^{-2}) was quantified for one leaf per stratum by taking an impression of the middle abaxial portion of the leaf with diluted clear nail enamel [43]. A 1 mm^{2} section of each impression was used to count stomata present through a compound microscope (10x magnification). The three leaves from each stratum were then combined for analysis of LN (mg g^{-1}) and leaf carbon to N ratio (CN) using a LECO CHN elemental analyzer. On each coffee leaf, CLR severity was quantified as the percent area of leaf with visible chlorotic lesions [44], determined using ImageJ software.

Statistical analyses

All statistical analyses were performed in RStudio version 1.1.456 (R Foundation for Statistical Computing, Vienna, Austria). We first calculated means and standard errors for all leaf traits and CLR severity and used a two-way analysis of variance (ANOVA) with a Tukey's post-hoc test to compare trait and CLR means among the shade tree canopy treatments and coffee plant stratum (where n = 519 leaves for all variables in each plant stratum, except for stomatal density and leaf chemical traits where n = 173). We then examined bivariate relationships among all leaf traits and CLR severity across all shade tree treatments, but focusing only on the middle stratum (where n = 519 for all models, except for stomatal density and leaf chemical traits where n = 173) using standardized major axis (SMA) regression models implemented in the 'lmodel2' R package [45]. CLR severity, leaf thickness, SLA, LDMC and CN were not normally distributed and were therefore log-transformed prior to analysis.

To understand whether or not relationships between coffee leaf functional traits and CLR severity differ between different shade tree canopy treatments and coffee plant strata, we used a hierarchical clustering analysis implemented in the 'dendextend' R package [46]. Specifically,

this analysis first determined the hierarchical clustering of coffee leaf traits and CLR severity using 'Ward's' method. In a dendrogram, each tip represents an individual leaf, where tips further apart represent leaves that are more dissimilar in their traits. We limited the traits to those with significant relationships with CLR (i.e., removing stomatal density). Cluster dendrograms for each trait-by-trait combination were aligned and compared using the 'step1side' method, from which an entanglement score was then calculated. In this analysis, a higher entanglement score indicates greater dissimilarity of the clusters, and a lower score indicates a better alignment or tighter relation between the clusters.

We then merged our leaf trait data from only the middle canopy stratum, with leaf trait data from previous work [21] on non-diseased Caturra plants, to test for differences in ITV relationships between diseased and healthy leaves. This published data is from the same coffee plant stratum (the middle stratum), cultivar, similar management regimes, the same region, and was collected at the same time of year, making this arguably the most comparable dataset on coffee leaf traits available. However, this data (n = 192) was from strictly healthy coffee plants with no signs of foliar disease [21]. To test for potential differences in bivariate leaf trait relationships between leaves with and without foliar disease, we used SMA and a linear hypothesis test using the 'smatr' package in R [47], to compare the slope and intercept of relationships between SLA-LN, and SLA-CN.

This merged dataset, including diseased and non-diseased trait values, was then further analyzed using principal components analysis (PCA) with the 'vegan' package in R [48]. Our PCA included leaf thickness, leaf area, SLA, LN and leaf CN, in order to include data that was present in both datasets. PCA results were grouped according to presence or absence of visible foliar disease, where n = 519 for leaves with CLR and n = 192 for those without. A

permutational multivariate analysis of variance (PERMANOVA) implemented with the 'adonis' function in the 'vegan' R package [48] was used to test for differences in multivariate trait variation between the two groupings (with 999 permutations used).

Results

Intraspecific trait variation of coffee leaves under diverse canopies

Coffee leaf traits differed significantly between both the shade tree canopy treatments and coffee plant strata (Table 1). However, since there were no consistent significant differences in leaf trait values across other levels of the nested experimental design (i.e., among blocks, amendments, or plant yield class), these were not explored in subsequent analyses. Morphological traits varied significantly between different shade tree treatments; in the middle canopy stratum, SLA was highest (20.4 ± 0.4 mg mm^{-2}) and LDMC lowest (235.7 ± 2.5 mg g^{-1}) in the Legume 1 + Legume 2 treatment. In contrast, SLA was lowest (16.3 ± 0.2 mg mm^{-2}) and LDMC the highest (262.0 ± 3.4 mg g^{-1}) in the Legume 1 treatment. Chemical traits varied according to the presence or absence of Legume 1 (*E. poeppigiana*), such that LN was significantly higher and CN significantly lower when Legume 1 was present across all coffee plant strata (ANOVA $P < 0.001$, Table 1).

Table 1. Means and standard errors of coffee leaf rust severity (CLR) and leaf traits in *Coffea arabica* across shade tree canopy treatments and coffee plant strata.

	Legume 1			Timber			Legume 2			Legume 1 + Timber			Legume 1 + Legume 2		
	Top	Middle	Bottom	Top	Middle	Bottom	Top	Middle	Bottom	Top	Middle	Bottom	Top	Middle	Bottom
CLR	0.5^{bB} ±0.1	0.8^{aB} ±0.1	0.7^{abB} ±0.1	1.2^{bA} ±0.2	1.7^{abB} ±0.4	2.7^{aA} ±0.4	1.2^{bA} ±0.2	3.6^{aA} ±0.7	2.6^{abA} ±0.7	0.5^{bB} ±0.1	1.1^{aB} ±0.2	0.8^{abB} ±0.1	0.5^{B} ±0.1	0.8^{B} ±0.1	0.8^{B} ±0.2
T	0.25^{a} ±0.003	0.24^{bB} ±0.002	0.22^{cB} ±0.002	0.25^{a} ±0.002	0.25^{bA} ±0.002	0.23^{bA} ±0.002	0.24^{a} ±0.002	0.24^{aB} ±0.003	0.22^{bB} ±0.002	0.25^{a} ±0.002	0.24^{bB} ±0.002	0.23^{cAB} ±0.002	0.25^{a} ±0.003	0.24^{abAB} ±0.002	0.23^{bAB} ±0.002
S	139.8^{B} ±5.5	133.6 ±4.4	123.9 ±5.3	164.1^{aA} ±6.8	149.1^{ab} ±8.2	131.9^{b} ±5.0	133.7^{B} ±4.6	139.5 ±5.4	129.0 ±5.0	131.2^{B} ±4.7	126.9 ±8.7	123.0 ±3.6	125.3^{abB} ±4.4	138.7^{a} ±5.1	113.7^{b} ±4.2
LA	37.5^{BC} ±1.2	41.3^{BC} ±1.4	39.6^{C} ±1.2	34.6^{bC} ±1.3	39.7^{aC} ±1.4	38.7^{aC} ±1.1	39.4^{B} ±1.2	39.1^{C} ±1.1	40.4^{BC} ±1.4	47.0^{A} ±1.3	47.0^{A} ±1.3	45.6^{AB} ±1.5	50.1^{aA} ±1.6	45.3^{bAB} ±1.3	49.5^{abA} ±1.5
SLA	15.0^{cB} ±0.3	16.3^{bB} ±0.2	18.2^{aC} ±0.3	15.7^{bB} ±0.5	16.8^{aB} ±0.3	17.2^{aC} ±0.3	17.3^{cA} ±0.3	18.8^{bA} ±0.4	20.0^{aB} ±0.4	17.1^{bA} ±0.3	19.7^{aA} ±0.3	19.8^{aB} ±0.3	18.0^{bA} ±0.3	20.4^{aA} ±0.4	21.6^{aA} ±0.5
LDMC	276.0^{aA} ±4.3	262.0^{bA} ±3.4	253.7^{bA} ±3.6	276.7^{aA} ±5.0	257.1^{bAB} ±4.5	255.8^{bA} ±4.6	250.8^{aC} ±3.8	241.8^{abC} ±3.9	233.4^{bB} ±4.6	268.3^{aAB} ±3.4	244.5^{bBC} ±3.0	248.0^{bAB} ±3.3	251.0^{aBC} ±3.3	235.7^{bC} ±2.5	237.0^{bB} ±3.7
LN	3.25^{bB} ±0.03	3.30^{abB} ±0.03	3.38^{aA} ±0.03	2.95^{bD} ±0.04	3.16^{aC} ±0.04	3.03^{abC} ±0.04	3.07^{bC} ±0.02	3.14^{abC} ±0.02	3.21^{aB} ±0.03	3.24^{bB} ±0.03	3.31^{abAB} ±0.03	3.37^{aA} ±0.03	3.40^{A} ±0.03	3.41^{A} ±0.02	3.44^{A} ±0.03
CN	14.13^{aC}	13.82^{abB}	13.43^{bC}	15.85^{aA}	14.67^{bA}	15.30^{abA}	15.01^{aB}	14.61^{abA}	14.26^{bB}	14.09^{aC}	13.74^{abBC}	13.48^{bC}	13.41^{D}	13.29^{C}	13.07^{C}

	±0.15	±0.13	±0.16	±0.25	±0.18	±0.24	±0.13	±0.12	±0.15	±0.12	±0.13	±0.15	±0.13	±0.11	±0.14

T = leaf thickness; S = stomatal density; LA = leaf area; SLA = specific leaf area; LDMC = leaf dry matter content; LN = leaf nitrogen concentration; CN = leaf carbon to nitrogen ratio; Lower case letters beside mean values denote significant differences ($P < 0.05$) between strata within a single shade tree treatment, whereas upper case letters denote significant differences ($P < 0.05$) between shade tree treatments within a single stratum, as determined by a two-way ANOVA.

Across all shade tree treatments, SLA (16.66 ± 0.15 mg mm^{-2}) and LN (3.19 ± 0.01 mg g^{-1}) were lowest and LDMC (264.12 ± 1.83 mg g^{-1}) and CN (14.44 ± 0.08) were highest in the top coffee stratum ($P < 0.001$). Only under Legume 1 + Legume 2 were there no significant differences in LN and CN across strata. CLR severity was lower in the top stratum and higher in the middle stratum, except for under Timber, where CLR severity was highest in the bottom stratum (Table 1).

Leaf trait co-variation and disease severity

In the middle canopy stratum, and across all shade tree treatments, we found evidence of coordinated trait relationships and trait co-variation with CLR severity (Table 2). Specifically, we found both greater SLA and LN were related to lower CLR severity ($r^2 = 0.03$, $P < 0.001$, and $r^2 = 0.078$, $P < 0.001$, respectively), whereas greater LDMC and CN were related to greater CLR severity ($r^2 = 0.019$, $P = 0.002$, and $r^2 = 0.059$, $P < 0.001$, respectively). Relationships between traits and CLR were robust when analyzing each canopy plant stratum individually, except for the top stratum where SLA and LDMC were not significantly related to CLR (data not shown).

Table 2. Bivariate standardized major axis regression analyses among coffee leaf rust severity (CLR) and leaf traits in *Coffea arabica* across all shade tree treatments in only the middle stratum.

	Disease metric	Morphological leaf traits					Chemical leaf traits	
	log CLR	log T	S	LA	log SLA	log LDMC	LN	log CN
log CLR	--	6.56<br>(6.02, 7.15)	-0.016<br>(-0.019, -0.014)	-0.050<br>(-0.054, -0.046)	-3.57<br>(-3.89, -3.28)	4.52<br>(4.15, 4.92)	-2.24<br>(-2.43, -2.06)	6.55<br>(6.02, 7.12)
log T	**0.02**<br>**(<0.001)**	--	-2.60E-3<br>(-0.003, -0.002)	0.008<br>(0.007, 0.008)	-0.54<br>(-0.59, -0.50)	0.69<br>(0.63, 0.75)	0.34<br>(0.31, 0.37)	-1.00<br>(-1.09, -0.92)
S	0.006<br>(0.304)	0.004<br>(0.404)	--	-2.90<br>(-3.35, -2.50)	-215.96<br>(-250.92, -185.86)	296.36<br>(256.16, 342.87)	-129.75<br>(-150.57, -111.80)	379.29<br>(327.04, 439.90)
LA	**0.017**<br>**(0.003)**	**0.054**<br>**(<0.001)**	**0.046**<br>**(0.005)**	--	71.98<br>(66.14, 78.34)	-91.11<br>(-99.26, -83.63)	45.15<br>(41.56, 49.06)	-131.95<br>(-143.32, -121.48)
log SLA	**0.030**<br>**(<0.001)**	**0.101**<br>**(<0.001)**	0.004<br>(0.390)	**0.038**<br>**(<0.001)**	--	-1.27<br>(-1.35, -1.180	0.63<br>(0.58, 0.68)	-1.83<br>(-1.99, -1.69)

log LDMC	**0.019**	0.006	**0.061**	**0.014**	**0.406**	--	-0.50	1.45
	**(0.002)**	(0.080)	**(0.001)**	**(0.007)**	**(<0.001)**		(-0.54, -0.46)	(1.34, 1.57)
LN	**0.078**	0.004	0.020	**0.076**	**0.088**	**0.186**	--	-2.92
	**(<0.001)**	(0.142)	(0.062)	**(<0.001)**	**(<0.001)**	**(<0.001)**		(-2.97, -2.88)
log CN	**0.059**	0.006	**0.029**	**0.082**	**0.074**	**0.185**	**0.970**	--
	**(<0.001)**	(0.078)	**(0.026)**	**(<0.001)**	**(<0.001)**	**(<0.001)**	**(<0.001)**	

Model r^2 and *P*-values (in brackets) for each bivariate model are presented in the lower left section of the matrix, with significant relationships ($P < 0.05$) highlighted in bold. The upper right section of the matrix presents model slopes and associated 95% confidence intervals (in brackets).

Strength of trait-disease relationships

Our hierarchical clustering analyses and entanglement scores indicated that differences in the strength of relationships between CLR severity and six leaf traits (specifically leaf thickness, leaf area, SLA, LDMC, LN, CN). Across each stratum, and across all shade tree treatments, entanglement scores ranged nearly 2-fold from 0.402 to 0.709 (Fig 2). Across all shade treatments pooled together, there was stronger alignment between traits and CLR severity in the middle stratum, except for Legume 1, where the top stratum had the strongest alignment (Fig 2).

Fig 2. Paired dendrograms of coffee leaf traits and CLR severity by plant strata for *Coffea arabica* leaves from each of the shade tree treatments. Entanglement scores are listed below each paired dendrogram. Euclidean distances were clustered using Ward's method. Paired dendrograms were first untangled to find the best alignment layout.

Differences in diseased and healthy environments

Patterns of coffee trait co-variation differed significantly between diseased and healthy environments (Fig 3). Using leaf trait data from only the middle coffee plant stratum, trait relationships shifted in relation to the presence or absence of CLR. Specifically, we found that bivariate relationships between SLA and LN was stronger in diseased leaves (SMA r^2= 0.088; $P < 0.001$) with a significantly steeper slope (0.63 ± 0.02; $P = 0.006$) and larger intercept (0.85 ± 0.03; $P < 0.001$) compared with healthy leaves (SMA $r^2 = 0.024$; $P < 0.001$; slope = 0.56 ± 0.03; intercept = 0.43 ± 0.05) (Fig 3). Similarly, we observed a stronger relationship between SLA and CN in diseased leaves (SMA $r^2 = 0.074$; $P < 0.001$) with a significantly greater intercept (7.73 ±

0.20; $P = 0.003$) compared to healthy leaves (SMA $r^2 = 0.041$; $P = 0.005$; intercept = 7.11 ± 0.33).

Fig 3. Bivariate trait relationships between (**a**) log-transformed specific leaf area (SLA) and leaf nitrogen concentration (LN); and (**b**) log-transformed SLA and log-transformed leaf carbon to nitrogen ratio (CN) of *Coffea arabica* leaves from the middle coffee plant stratum in shaded agroecosystems, where $n = 519$ for diseased leaves and $n = 192$ for healthy leaves.

Principal component analysis (PCA) of our merged dataset of leaves with and without visible signs of foliar disease, indicated that the first two PCA axes explain a total of 70.4% of the variation in the coffee leaf functional traits included (Fig 4). PCA axis 1 explained 40.7% of variation in multivariate traits being significantly positively associated with LN ($r^2 = 0.835$, $P < 0.001$), leaf area ($r^2 = 0.204$, $P < 0.001$) and SLA ($r^2 = 0.068$, $P < 0.001$), and negatively associated with CN ($r^2 = 0.923$, $P < 0.001$). PCA axis 2 explained an additional 29.7% of the variation in traits and was significantly positively associated with leaf thickness ($r^2 = 0.741$, $P < 0.001$) and LN ($r^2 = 0.045$, $P < 0.001$), and negatively associated with SLA ($r^2 = 0.687$, $P < 0.001$). PERMANOVA indicated that leaf traits in our merged dataset differed significantly between healthy and diseased environments ($F = 124.86$, $P < 0.001$), such that diseased leaves expressed greater SLA, whereas healthy leaves shifted towards greater leaf thickness.

Fig 4. Principal component analysis of five coffee leaf functional traits (leaf thickness (T); leaf area (LA); specific leaf area (SLA); leaf nitrogen concentration (LN); leaf carbon to nitrogen ratio (CN)) from the middle stratum of *Coffea arabica* leaves. Symbols represent presence or

absence of CLR (where $n = 519$ for diseased leaves and $n = 192$ for healthy leaves). Circles correspond to 95% confidence ellipses for coffee leaves sampled within each category, where the solid line represents the healthy grouping, and the dashed line represents the diseased grouping.

Discussion

Intra-plant trait variability

While multiple studies have documented intraspecific trait variation in coffee and other wild and domesticated plant species, e.g. [2,25,49], our results demonstrate for the first time that ITV and trait relationships are maintained in leaves with visible signs of foliar disease. Furthermore, we found evidence of intra-plant trait variability across three coffee plant strata independent of shade tree treatment, which highlights the need of this critical consideration when reporting trait mean values to characterize an entire plant. Specifically, we found that leaves from the bottom stratum expressed more resource acquiring trait values (i.e., high SLA coupled with high LN), whereas leaves from the top stratum expressed resource conserving trait values (i.e., high LDMC coupled with high leaf CN). This is similar to findings by [50], who found that leaf area, SLA and LN were highest in the bottom coffee plant stratum.

~~This~~ stratification was also evident in the alignment of key coffee leaf functional traits and CLR severity through hierarchical clustering. We found that coffee leaf trait clusters (specifically based on leaf thickness, leaf area, SLA, LDMC, LN, CN) were more strongly aligned with CLR severity in the middle stratum; a finding that was consistent across all but one shade tree treatment. These patterns can be the result of the interacting role of light availability on coffee leaf trait expression. In coffee, leaf trait plasticity in part is driven by the heterogeneity of light transmittance [18], which leads to greater resource acquiring trait values in light-limited

environments [25], and/or self-shaded leaves [51]. These patterns can also be a consequence of differences in intra-plant uredospore deposition patterns, where more spores are deposited on lower coffee plant strata [52], leading to greater CLR severity in lower strata, as observed in our study. Quantifying these complex trait-environment-pathogen interactions are therefore vital in understanding and modelling pathological dynamics in agroecosystems.

Leaf functional traits and foliar disease

While CLR incidence depends on the amount of inoculum present in a given area [53], the severity to which individual coffee leaves are affected by CLR depends on foliar traits that could change in response to, or interaction with, the pathogen. We hypothesized a positive relationships between stomatal density and leaf susceptibility to CLR, due to the increased probability of uredospores penetrating through stomatal openings [28]. However, in our study we did not find evidence of a significant relationship between CLR severity and stomatal density, with little variability of stomatal density across the different shade tree treatments and coffee plant strata. Therefore, CLR severity is likely more closely related to actual stomatal functioning (i.e., stomatal opening), as it is the opening of stomata that promotes the increased release of sporogenous pathogen cells [54]. In this case, variability in stomatal opening and pathogen behaviour has been related to key microclimate variability with specific timing in the pathogen’s lifecycle [32].

In the middle coffee canopy stratum, we found that more resource conservative trait values (i.e., high LDMC and high leaf CN values) were positively correlated with CLR severity, whereas greater resource acquisitive trait values (i.e., high SLA and high LN values) were significantly negatively correlated across all levels of the nested experimental design,

contradicting our second hypothesis. Based on previous research, one would expect that leaves with more resource acquisitive trait values would have greater CLR severity, as larger leaf area is thought to increase the probability of *H. vastatrix* uredospore interception [28], and studies have demonstrated a positive correlation between SLA and disease severity in other plant species [12,13], potentially due to thinner cell walls and more non-structural carbohydrates aiding biotrophic fungal pathogens [12]. When considering nutrient-based traits, there are more diverging results in the literature. For example, high N supply (via fertilization of the soil) combined with low light (similar to the shaded conditions of our study), presumably leading to greater foliar N content, resulted in slower rates of infection and sporulation of *H. vastatrix* in *C. arabica* [55]. In contrast, limited soil N availability for wheat resulted in reduced spore production of leaf rust (*Puccinia triticina*) due to less efficient sporulation [56]. In Douglas-fir infected with the biotrophic fungus *Phaeocryptopus gaeumannii*, high LN is correlated with high disease severity, likely due to greater nutrient availability for the pathogen within the apoplast, where *P. gaeumannii* resides, and from potential cell membrane leakage [15]. These contrasting results are expected, as the effects of foliar nutrient availability on disease severity is pathogen-specific, dictating both the level of defensive compound production and the metabolic activity of host cells [14,57].

While individual relationships between traits and disease severity provide some insight into potential constitutive or induced defensive mechanisms, considering traits together as trait syndromes better reflects plant resource use strategies specific to the plant genotype in question. Through this lens, our results suggest that the greater resource acquisitive traits, which together promote enhanced C assimilation rates in coffee [25], could lead to reduced plant stress and improved disease tolerance via enhanced compensatory growth [16,58]. Indeed, enhanced leaf

growth via higher gas exchange rates has been observed in other pathogen-tolerant plant genotypes, including Dutch elm hybrids [13] and spring barley cultivars [59]. Given that CLR severity was measured as the percent leaf area with visible chlorotic lesions [44], enhanced leaf growth can lead to a dilution effect [60], reducing the magnitude of CLR severity measured.

Intraspecific leaf functional trait relationships in diseased environments

When comparing trait relationships in leaves with and without visible signs of foliar disease, we found that the shape of these relationships remained consistent, highlighting how CLR does not destabilize the nature of established global leaf trait relationships. However, despite these consistencies, there was an overall shift towards more resource acquiring trait values when foliar disease was apparent, confirming our third hypothesis. Based on our PCA results, coffee leaves that showed signs of CLR shifted significantly towards greater SLA values along PCA axis 2, compared to healthy coffee leaves with no signs of foliar disease that shifted towards greater leaf thickness. Similarly, key bivariate relationships between SLA with LN and CN shifted in elevation towards a greater SLA value for a given LN or CN value, in diseased compared to healthy leaves. As noted above, previous research has suggested that more resource acquiring traits, including high SLA, is linked with greater disease susceptibility [12,13], due to structural foliar differences [12]. Therefore, this shift towards resource acquisition in diseased leaves likely reflects a greater constitutive susceptibility to pathogen infection and development, rather than an induced response to pathogen infection [61].

Conclusion

Our results provide evidence that plant functional traits and their relationships shift under pathogen pressures ~~and~~ represent among the first lines of evidence indicating that ITV plays a role in mediating disease severity ~~in managed terrestrial ecosystems~~. In doing so, our work both advances our understanding of the functional ecology of a major crop pathogen and addresses a vital gap in our ~~collective~~ understanding of global patterns of functional trait variation~~,~~ ~~highlighting the key role that~~ ITV plays in ~~governing~~ plant-pathogen-environment interactions ~~and the need to further explore pathogens within the global functional trait space~~

Acknowledgements

The authors sincerely thank L. Romero and V. H. Mendez Sanabria for their invaluable assistance in the field. We would also like to thank the anonymous reviewers for reading earlier versions of this paper and providing helpful and constructive comments. This work was supported by the Natural Sciences and Engineering Research Council of Canada (Discovery Grant to M.E.I. and Alexander Graham Bell Canada Graduate Scholarship to S.G.).

Data Availability

Data are archived in the TRY functional trait database at try-db.org.

References

1. Wright IJ, Westoby M, Reich PB, Oleksyn J, Ackerly DD, Baruch Z, et al. The worldwide leaf economics spectrum. Nature. 2004;428: 821–827. doi:10.1038/nature02403
2. Albert CH, Thuiller W, Yoccoz NG, Soudant A, Boucher F, Saccone P, et al. Intraspecific functional variability: Extent, structure and sources of variation. J Ecol. 2010;98: 604–

613. doi:10.1111/j.1365-2745.2010.01651.x

3. Reich PB. The world-wide "fast-slow" plant economics spectrum: A traits manifesto. J Ecol. 2014;102: 275–301. doi:10.1111/1365-2745.12211

4. Ordoñez JC, Van Bodegom PM, Witte JPM, Wright IJ, Reich PB, Aerts R. A global study of relationships between leaf traits, climate and soil measures of nutrient fertility. Glob Ecol Biogeogr. 2009;18: 137–149. doi:10.1111/j.1466-8238.2008.00441.x

5. Díaz S, Kattge J, Cornelissen JHC, Wright IJ, Lavorel S, Dray S, et al. The global spectrum of plant form and function. Nature. 2016;529: 167–171. doi:10.1038/nature16489

6. Cadotte MW. The new diversity: Management gains through insights into the functional diversity of communities. J Appl Ecol. 2011;48: 1067–1069. doi:10.1111/j.1365-2664.2011.02056.x

7. Cui E, Weng E, Yan E, Xia J. Robust leaf trait relationships across species under global environmental changes. Nat Commun. 2020;11: 1–9. doi:10.1038/s41467-020-16839-9

8. Pérez-Harguindeguy N, Díaz S, Garnier E, Lavorel S, Poorter H, Jaureguiberry P, et al. New handbook for standardised measurement of plant functional traits worldwide. Aust J Bot. 2016;64: 715. doi:10.1071/BT12225_CO

9. Cornelissen JHC, Lavorel S, Garnier E, Díaz S, Buchmann N, Gurvich DE, et al. A handbook of protocols for standardised and easy measurement of plant functional traits worldwide. Aust J Bot. 2003;51: 335–380. doi:10.1071/BT02124

10. Gregory PJ, Johnson SN, Newton AC, Ingram JSI. Integrating pests and pathogens into the climate change/food security debate. J Exp Bot. 2009;60: 2827–2838. doi:10.1093/jxb/erp080

11. Kergunteuil A, Descombes P, Glauser G, Pellissier L, Rasmann S. Plant physical and chemical defence variation along elevation gradients: A functional trait-based approach. Oecologia. 2018;187: 561–571. doi:10.1007/s00442-018-4162-y

12. Toome M, Heinsoo K, Luik A. Relation between leaf rust (*Melampsora epitea*) severity and the specific leaf area in short rotation coppice willows. Eur J Plant Pathol. 2010;126: 583–588. doi:10.1007/s10658-009-9566-4

13. Ďurkovič J, Čaňová I, Lagaňa R, Kučerová V, Moravčík M, Priwitzer T, et al. Leaf trait dissimilarities between Dutch elm hybrids with a contrasting tolerance to Dutch elm disease. Ann Bot. 2013;111: 215–227. doi:10.1093/aob/mcs274

14. Walters DR, Bingham IJ. Influence of nutrition on disease development caused by fungal pathogens: Implications for plant disease control. Ann Appl Biol. 2007;151: 307–324. doi:10.1111/j.1744-7348.2007.00176.x

15. El-Hajj Z, Kavanagh K, Rose C, Kanaan-Atallah Z. Nitrogen and carbon dynamics of a foliar biotrophic fungal parasite in fertilized Douglas-fir. New Phytol. 2004;163: 139–147. doi:10.1111/j.1469-8137.2004.01102.x

16. Bingham IJ, Walters DR, Foulkes MJ, Paveley ND. Crop traits and the tolerance of wheat and barley to foliar disease. Ann Appl Biol. 2009;154: 159–173. doi:10.1111/j.1744-7348.2008.00291.x

17. Fagard M, Launay A, Clément G, Courtial J, Dellagi A, Farjad M, et al. Nitrogen metabolism meets phytopathology. J Exp Bot. 2014;65: 5643–5656. doi:10.1093/jxb/eru323

18. Matos FS, Wolfgramm R, Gonç F V, Cavatte PC, Ventrella MC, Damatta FM. Phenotypic plasticity in response to light in the coffee tree. Environ Exp Bot. 2009;67: 421–427.

doi:10.1016/j.envexpbot.2009.06.018

19. DaMatta FM, Ramalho JDC. Impacts of drought and temperature stress on coffee physiology and production: A review. Brazilian J Plant Physiol. 2006;18: 55–81. doi:10.1590/S1677-04202006000100006

20. DaMatta FM, Loos RA, Silva EA, Loureiro ME. Limitations to photosynthesis in *Coffea canephora* as a result of nitrogen and water availability. J Plant Physiol. 2002;159: 975–981. doi:10.1078/0176-1617-00807

21. Martin AR, Rapidel B, Roupsard O, Van den Meersche K, Virginio Filho E de M, Barrios M, et al. Intraspecific trait variation across multiple scales: The leaf economics spectrum in coffee. Funct Ecol. 2017;31: 604–612. doi:10.1111/1365-2435.12790

22. Fulthorpe R, Martin AR, Isaac ME. Root endophytes of coffee (*Coffea arabica*): Variation across climatic gradients and relationships with functional traits. Phytobiomes J. 2020;4: 27–39. doi:10.1094/PBIOMES-04-19-0021-R

23. Martin AR, Isaac ME. The leaf economics spectrum's morning coffee: Plant size-dependent changes in leaf traits and reproductive onset in a perennial tree crop. Ann Bot. 2021;127: 483–493. doi:10.1093/aob/mcaa199

24. Buchanan S, Isaac ME, Van den Meersche K, Martin AR. Functional traits of coffee along a shade and fertility gradient in coffee agroforestry systems. Agrofor Syst. 2019;93: 1261–1273. doi:10.1007/s10457-018-0239-1

25. Gagliardi S, Martin AR, Virginio Filho E de M, Rapidel B, Isaac ME. Intraspecific leaf economic trait variation partially explains coffee performance across agroforestry management regimes. Agric Ecosyst Environ. 2015;200: 151–160. doi:10.1016/j.agee.2014.11.014

26. Hayes FJ, Buchanan SW, Coleman B, Gordon AM, Reich PB, Thevathasan N V., et al. Intraspecific variation in soy across the leaf economics spectrum. Ann Bot. 2019;123: 107–120. doi:10.1093/aob/mcy147

27. Kushalappa AC, Eskes AB. Advances in coffee rust research. Annu Rev Phytopathol. 1989;27: 503–531. doi:10.1146/annurev.py.27.090189.002443

28. Avelino J, Willocquet L, Savary S. Effects of crop management patterns on coffee rust epidemics. Plant Pathol. 2004;53: 541–547. doi:10.1111/j.1365-3059.2004.01067.x

29. Talhinhas P, Batista D, Diniz I, Vieira A, Silva DN, Loureiro A, et al. The coffee leaf rust pathogen Hemileia vastatrix: One and a half centuries around the tropics. Mol Plant Pathol. 2017;18: 1039–1051. doi:10.1111/mpp.12512

30. Gagliardi S, Avelino J, Virginio Filho E de M, Isaac ME. Shade tree traits and microclimate modifications: Implications for pathogen management in biodiverse coffee agroforests. Biotropica. 2021;53: 1356–1367. doi:10.1111/btp.12984

31. Gagliardi S, Avelino J, Beilhe LB, Isaac ME. Contribution of shade trees to wind dynamics and pathogen dispersal on the edge of coffee agroforestry systems: A functional traits approach. Crop Prot. 2020;130: 105071. doi:10.1016/j.cropro.2019.105071

32. Merle I, Tixier P, Virginio Filho E de M, Cilas C, Avelino J. Forecast models of coffee leaf rust symptoms and signs based on identified microclimatic combinations in coffee-based agroforestry systems in Costa Rica. Crop Prot. 2020;130: 105046. doi:10.1016/j.cropro.2019.105046

33. Loranger J, Shipley B. Interspecific covariation between stomatal density and other functional leaf traits in a local flora. Botany. 2010;88: 30–38. doi:10.1139/B09-103

34. Silva M do C, Nicole M, Guerra-Guimara L, Ro CJ. Hypersensitive cell death and post-

haustorial defence responses arrest the orange rust (*Hemileia vastatrix*) growth in resistant coffee leaves. Physiol Mol Plant Pathol. 2002;60: 169–183. doi:10.1006/pmpp.2002.0389

35. Avelino J, Zelaya H, Merlo A, Pineda A, Ordõ Nez M, Savary S. The intensity of a coffee rust epidemic is dependent on production situations. Ecol Modell. 2006;197: 431–447. doi:10.1016/j.ecolmodel.2006.03.013

36. Carvalho VL de, Chalfoun SM, Castro HA de, Carvalho VD de. Influence of different yield levels on coffee rust evolution and on phenolic compounds on leaves. Ciência e Agrotecnologia. 2001;25: 49–54.

37. Haggar J, Barrios M, Bolaños M, Merlo M, Moraga P, Munguia R, et al. Coffee agroecosystem performance under full sun, shade, conventional and organic management regimes in Central America. Agrofor Syst. 2011;82: 285–301. doi:10.1007/s10457-011-9392-5

38. Westerband AC, Funk JL, Barton KE. Intraspecific trait variation in plants: A renewed focus on its role in ecological processes. Ann Bot. 2021; 1–14. doi:10.1093/aob/mcab011

39. Tscharntke T, Clough Y, Bhagwat SA, Buchori D, Faust H, Hertel D, et al. Multifunctional shade-tree management in tropical agroforestry landscapes - A review. J Appl Ecol. 2011;48: 619–629. doi:10.1111/j.1365-2664.2010.01939.x

40. Upreti G, Bittenbender HC, Ingamells JL. Rapid estimation of coffee yield. Proceedings of the Fourteenth International Scientific Colloquium on Coffee. San Francisco; 1992. Available: http://asic-cafe.org/system/files/14_065.pdf

41. Audebert L. Productivité aérienne du caféier Agroforestier: effets des arbres d’ombrage et de l’âge des rejets. UHP Nancy I, Nancy, France. 2011. Available: http://orton.catie.ac.cr/repdoc/A11074f/A11074f.pdf

42. Messier J, McGill BJ, Lechowicz MJ. How do traits vary across ecological scales? A case for trait-based ecology. Ecol Lett. 2010;13: 838–848. doi:10.1111/j.1461-0248.2010.01476.x
43. Silva Acuña R, Silva Acuña A, Zambolim L. Indice estomático en materiales genéticos de café resistentes o susceptibles a la roya. Bioagro. 1998;10: 57–61.
44. Madden L V., Hughes G, van den Bosch F. Measuring plant diseases. In: Madden L V., Hughes G, van den Bosch F, editors. The Study of Plant Disease Epidemics. The American Phytopathological Society; 2017. pp. 11–31. doi:10.1094/9780890545058.002
45. Legendre P. lmodel2: Model II Regression. R package version 1.7-3. 2018. Available: https://cran.r-project.org/package=lmodel2
46. Galili T. dendextend: an R package for visualizing, adjusting and comparing trees of hierarchical clustering. Bioinformatics. 2015;31: 3718–3720. doi:10.1093/bioinformatics/btv428
47. Warton DI, Duursma RA, Falster DS, Taskinen S. smatr 3 - an R package for estimation and inference about allometric lines. Methods Ecol Evol. 2012;3: 257–259.
48. Oksanen J, Blanchet FG, Friendly M, Kindt R, Legendre P, McGlinn D, et al. vegan: Community ecology package. R package version 2.5-7. 2020. Available: https://cran.r-project.org/package=vegan
49. Martin AR, Hale CE, Cerabolini BEL, Cornelissen JHC, Craine J, Gough WA, et al. Inter- and intraspecific variation in leaf economic traits in wheat and maize. AoB Plants. 2018;10: 1–14. doi:10.1093/aobpla/ply006
50. Charbonnier F, Roupsard O, le Maire G, Guillemot J, Casanoves F, Lacointe A, et al. Increased light-use efficiency sustains net primary productivity of shaded coffee plants in

agroforestry system. Plant Cell Environ. 2017;40: 1592–1608. doi:10.1111/pce.12964

51. Butler DR. Coffee leaf temperatures in a tropical environment. Acta Bot Neerl. 1977;26: 129–140. doi:10.1111/j.1438-8677.1977.tb01106.x

52. Avelino J, Vílchez S, Segura-Escobar MB, Brenes-Loaiza MA, Virginio Filho E de M, Casanoves F. Shade tree Chloroleucon eurycyclum promotes coffee leaf rust by reducing uredospore wash-off by rain. Crop Prot. 2020;129: 105038. doi:10.1016/j.cropro.2019.105038

53. Merle I, Pico J, Granados E, Boudrot A, Tixier P, Virginio Filho E de M, et al. Unraveling the complexity of coffee leaf rust behavior and development in different *Coffea arabica* agroecosystems. Phytopathology. 2020;110: 418–427. doi:10.1094/PHYTO-03-19-0094-R

54. Hoogstraten JGJ, Toma-Braghini M, Eskes AB. Influencia da umidade do solo e umidade relativa do ar sobre a resistencia do cafeeiro a Hemileia vastatrix. Proceedings of the 10° Congresso Brasileiro de Pesquisas Cafeeiras. Poços de Caldas, Minas Gerais, Brasil; 1983.

55. Toniutti L, Breitler J-C, Etienne H, Campa C, Doulbeau S, Urban L, et al. Influence of environmental conditions and genetic background of Arabica coffee (*C. arabica* L) on leaf rust (*Hemileia vastatrix*) pathogenesis. Front Plant Sci. 2017;8: 2023. doi:10.3389/fpls.2017.02025

56. Robert C, Bancal M-O, Lannou C. Wheat leaf rust uredospore production on adult plants: Influence of leaf nitrogen content and Septoria tritici blotch. Phytopathology. 2004;94: 712–721. doi:10.1094/PHYTO.2004.94.7.712

57. Dordas C. Role of nutrients in controlling plant diseases in sustainable agriculture. A

review. Agron Sustain Dev. 2008;28: 33–46. doi:10.1051/agro

58. Schroth G, Krauss U, Gasparotto L, Aguilar JAD, Vohland K. Pests and diseases in agroforestry systems of the humid tropics. Agrofor Syst. 2000;50: 199–241. doi:https://doi.org/10.1023/A:1006468103914

59. Kral G, Oerke E-C, Schönbeck F. Influence of powdery mildew on the water balance and photosynthesis of spring barley cultivars differing in tolerance. J Plant Dis Prot. 1993;100: 1–10. Available: www.jstor.org/stable/43386133

60. Ferrandino FJ. Effect of crop growth and canopy filtration on the dynamics of plant disease epidemics spread by aerially dispersed spores. Phytopathology. 2008;98: 492–503. doi:10.1094/PHYTO-98-5-0492

61. Silva M do C, Várzea V, Guerra-Guimarães L, Azinheira HG, Fernandez D, Petitot A-S, et al. Coffee resistance to the main diseases: leaf rust and coffee berry disease. Brazilian J Plant Physiol. 2006;18: 119–147.

 Click here to access/download;Figure;Figure 1a.jpg

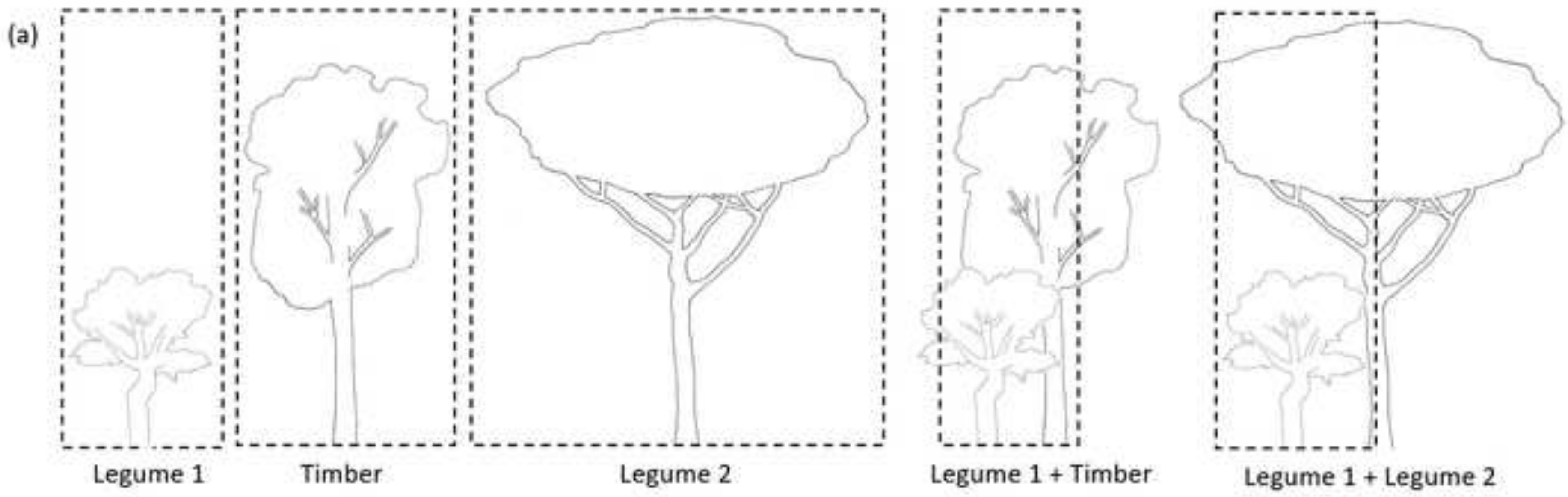

Figure 1b

Click here to access/download;Figure;Figure 1b.jpg

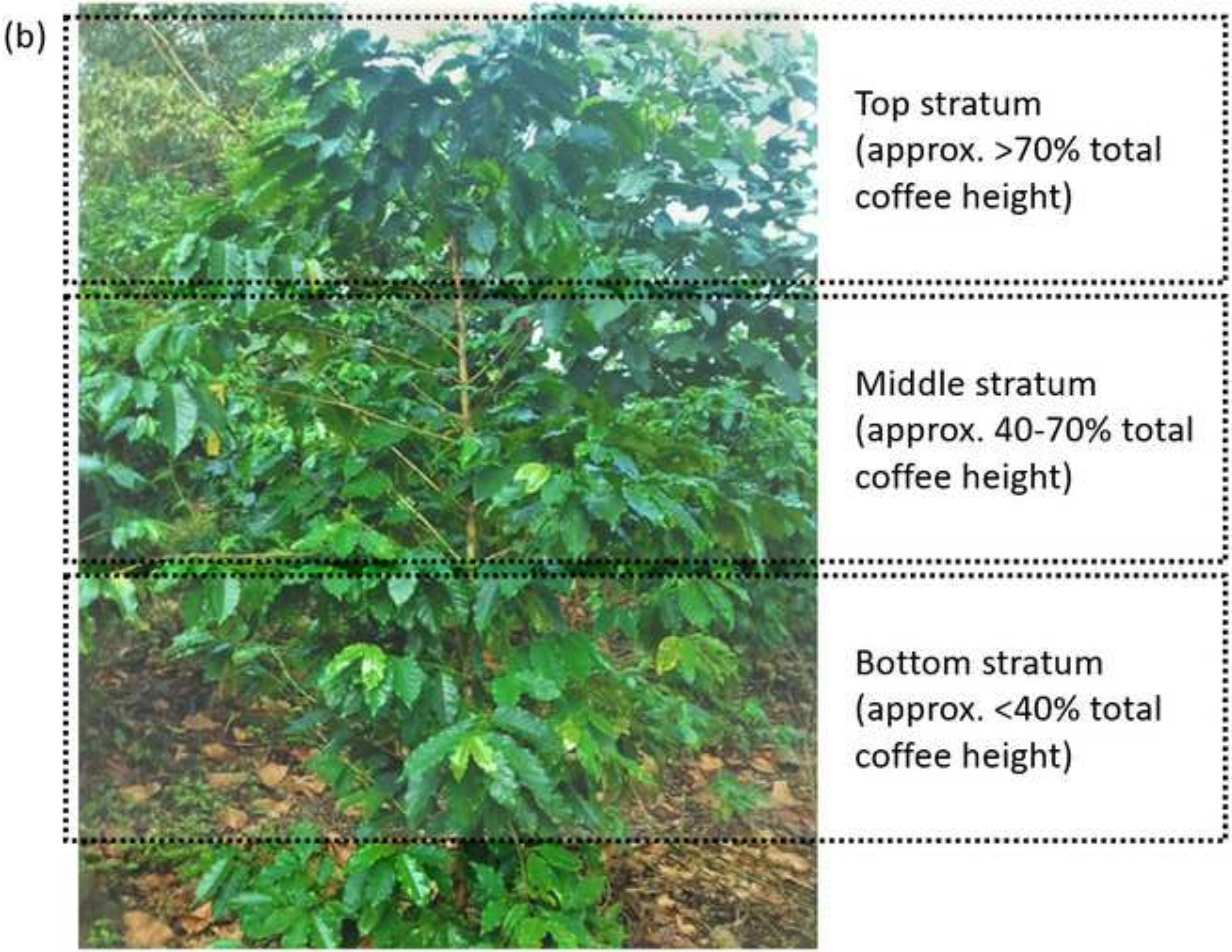

Figure 2

Click here to access/download;Figure;Figure 2.jpg

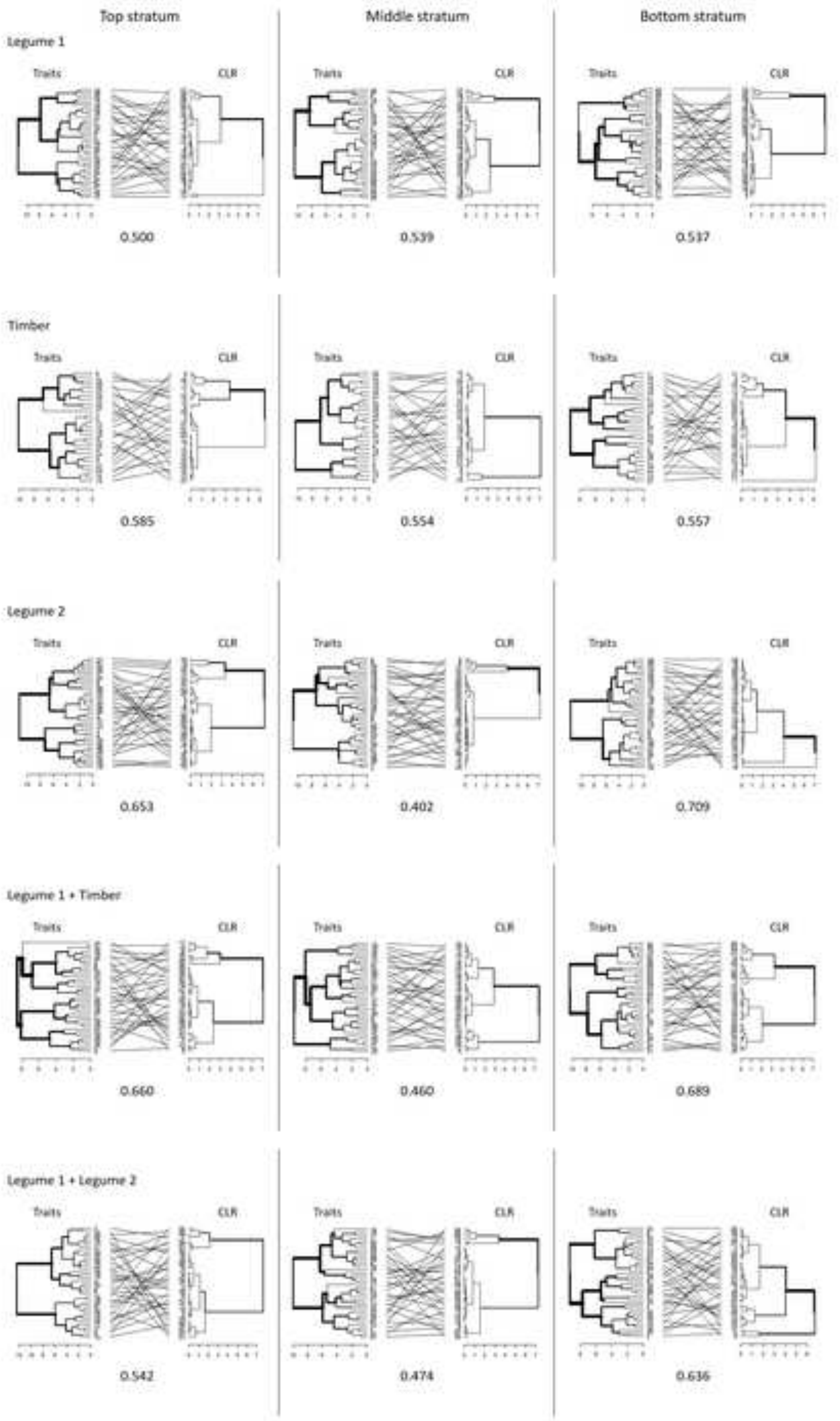

Figure 3

Click here to access/download;Figure;Figure 3.jpg

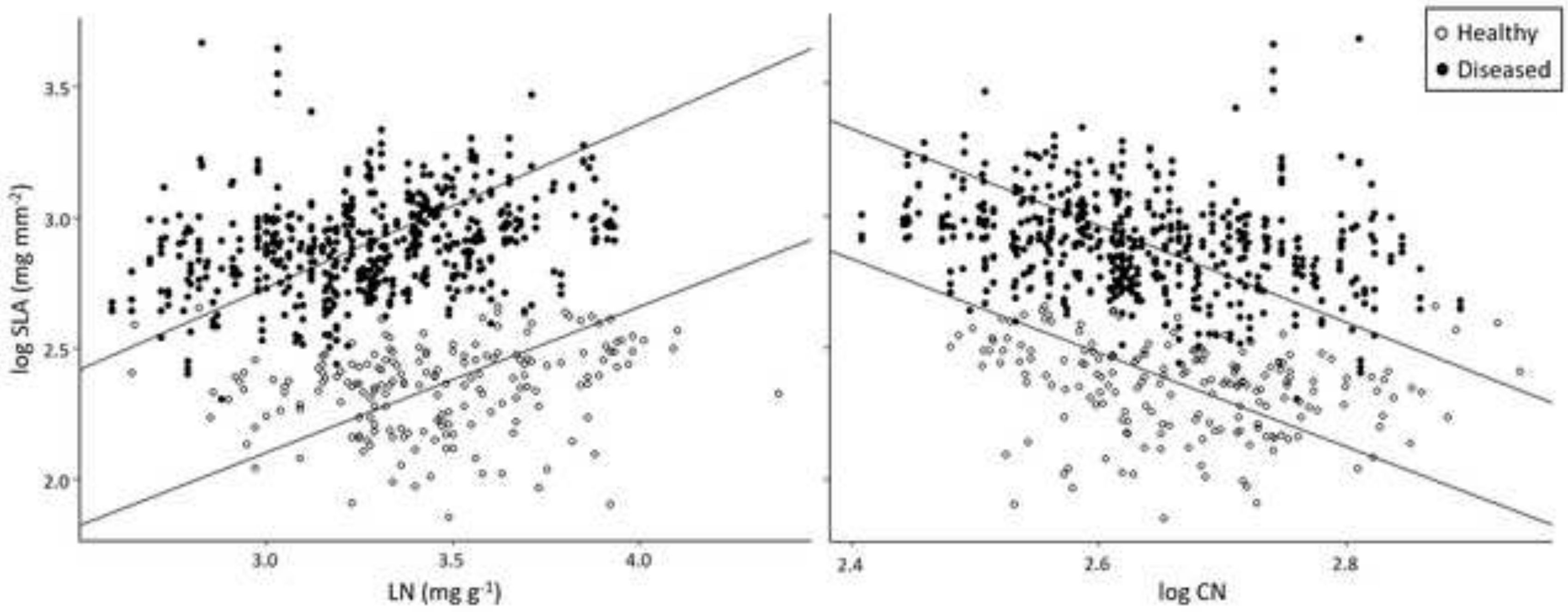

Figure 4

Click here to access/download;Figure;Figure 4.jpg

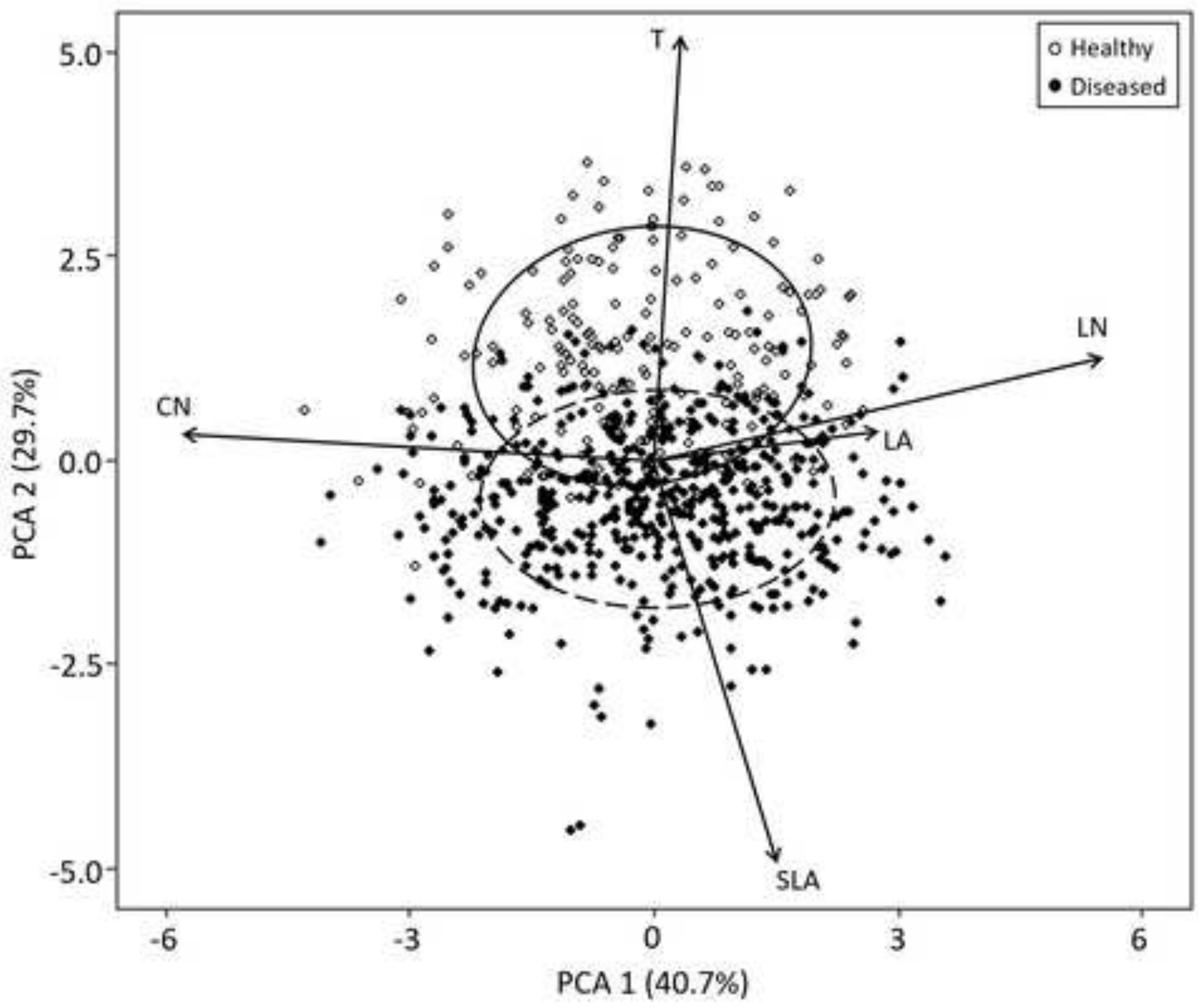

Supplement: Attachment — Submitted filename: PONE-D-22-31465_edits.pdf [file pone.0284203.s003.pdf]
