# Supplementary material for: Leaf functional traits and pathogens: Linking coffee leaf rust with intraspecific trait variation in diversified agroecosystems
Source: PLoS One. 2023 Apr 13;18(4):e0284203. doi: 10.1371/journal.pone.0284203 (PMC10101423; doi:10.1371/journal.pone.0284203)
Supplement: Attachment — Submitted filename: Response to Reviewers.docx [file pone.0284203.s004.docx]

Ref. no. PONE-D-22-31465

On behalf of my co-authors, we would like to thank you for reviewing our manuscript and we greatly appreciate the reviewers for their insightful comments and suggestions. We have revised our entire manuscript carefully and extensively, while following the reviewers’ suggestions. All changes are tracked in the “Revised Manuscript with Track Changes” file, and we provide detailed responses to each reviewer comment below. Where possible, we provide line numbers to refer to specific locations in the revised manuscript without tracked changes for simplicity (“Manuscript” file).

# Response to Reviewers

# Academic Editor

1. Please ensure that your manuscript meets PLOS ONE's style requirements, including those for file naming.

- Thank you for directing us to the appropriate style guides for our manuscript. We have made formatting changes throughout the manuscript to meet these guidelines and have also adjusted the file names as needed.

2. In your Methods section, please provide additional information regarding the permits you obtained for the work. Please ensure you have included the full name of the authority that approved the field site access and, if no permits were required, a brief statement explaining why.

- Since no permits were required for access to our field sites, we have added details to the “Materials and methods” section, lines 127-130, where we now state, “This study was conducted at the international coffee agroforestry research trial established by the Centro Agronómico Tropical de Investigación y Enseñanza (CATIE), located in Turrialba, Costa Rica (09°53´44” N, 83°40´7” W, 685 m a.s.l., with approval from and in collaboration with CATIE’s Trial Coordinator.”

3. Please include a complete copy of PLOS’ questionnaire on inclusivity in global research in your revised manuscript.

- The completed PLOS questionnaire on inclusivity in global research is now attached as a Supporting Information. We now state in the “Materials and methods” section, lines 130-132, “Additional information regarding the ethical, cultural, and scientific considerations specific to inclusivity in global research is included in the Supporting Information (S1 Checklist).”

4. Please remove any funding-related text from the manuscript and let us know how you would like to update your Funding Statement.

- We have not changed our Funding Statement. Our new Acknowledgements Statement (included in our cover letter) now reads, “The authors sincerely thank L. Romero and V. H. Mendez Sanabria for their invaluable assistance in the field, and the AGROFORESTA Scientific Platform for its support and collaboration with CATIE's Long-Term Trial with Agroforestry Systems in Cafe. We would also like to thank the anonymous reviewers for reading earlier versions of this paper and providing helpful and constructive comments.

5. If you wish to make changes to your Data Availability statement, please describe these changes in your cover letter and we will update your Data Availability statement to reflect the information you provide.

- No changes need to be made to our Data Availability statement at this time.

6. We note that you have included the phrase “data not shown” in your manuscript.

- We have now added the data as Supporting Information (in S2 Table), now cited in line 262.

7. Please review your reference list to ensure that it is complete and correct.

- Our reference list was revised to address Reviewer 2’s comments and to include more recently published literature on the topic. Otherwise, no references were removed due to retraction.

# Reviewer 1

1. Audacious title that does not reflect the experiment conducted..... I suggest modifying it for the evaluated pathosystem that is *Coffea arabica* x *Hemileia vastatrix*.

- Thank you for bringing up this point to make our title more specific. We have now added in the foliar disease we assess (coffee leaf rust) and changed the wording in our title (e.g., rather than “shapes”, we use “linking”). The title has now changed to “Leaf functional traits and pathogens: Linking coffee leaf rust with intraspecific trait variation in diversified agroecosystems”

2. Figure 2 should be redone.

- Given the poor image quality of Figure 2 in our original submission, we’ve re-uploaded the image file to be clearer.

3. The studied species *Erythrina poeppigiana, Chloroleucon eurycyclum,* and *Terminalia amazonia* are commonly used in intercropping with coffee trees by coffee growers????

- Indeed, intercropped tree species in coffee systems vary greatly around the world. To provide the reviewer with some clarification, we do not state that these specific species are commonly used (though *E. poeppigiana* is particularly common in the study region); rather, we state that the integration of timber and leguminous species in coffee systems is a common practice. To provide further clarification, we now state “The shade trees in this study included both timber and leguminous species, a common practice in coffee agroforestry systems in the study region of central Costa Rica” (lines 148-150).

4. With regard to the nutritional part, which has 14 nutrients, why did you discuss only nitrogen?

- This is a great question. Since the objective of our study is related to traits within the Leaf Economics Spectrum, we limited the analyses of chemical traits to reflect what is commonly reported in the functional trait literature. We now clarify this in lines 183-184 where we say, “Our analysis was restricted to these chemical traits to reflect what is commonly reported in the functional trait literature.”

5. Due to the large number of parameters, the manuscript as presented is confusing .... I suggest reformulation....

- Unfortunately, without details on which aspects of the manuscript are confusing or in which way, reformulation of the manuscript would be difficult and was not completed for these revisions.

# Reviewer 2

From a phytopathological view I have many open questions, and think the paper is too plant-centric and not giving enough credit on the active nature of plant parasites in manipulating plant species, coping with plant defense or plant traits… Nevertheless, I cannot keep myself from suggesting including the "pathogen view" in coming papers of this research topic more strongly.

- Overall, we agree that this is a plant-centric paper, given the focus is on leaf ITV. We had tried to allude to the active role of pathogens, and in our revised draft, we have made this clearer throughout the manuscript. For example:
  - Introduction, line 62, we now state “…despite emerging research linking the two and the co-evolution of many plant-pathogen systems.”
  - Model species, lines 103-104, we now state “… a foliar disease caused by *Hemileia vastatrix*, a biotrophic fungus that has co-evolved with *Coffea* spp., …”
  - Model species, lines 123-124, we now state “…despite the role trait expression may play in moderating plant-pathogen dynamics or the role that disease may play in manipulating common trait expression.”
  - Discussion, lines 373-376, we now state “These contrasting results are expected, given the unique nature of different plant-pathogen co-evolution strategies and, in turn, the pathogen-specific effects of foliar nutrient availability on disease, dictating both the level of defensive compound production and the metabolic activity of host cells.”
  - Conclusion, lines 411-412, we now state “…still only a glimpse into the response-effect co-evolution between plants and their native pathogens…”

## Minor changes

- We have accepted all minor wording changes throughout the manuscript as suggested in the pdf (and noted in the “Revised Manuscript with Track Changes” file), except for the following:
  - Abstract, line 33, it was recommended to change the wording from “key coffee leaf functional traits” to “key functional traits of coffee leaves”. We have decided to maintain the original wording, as this is the accepted wording in the functional trait literature (now line 29)
  - Introduction, line 61, it was recommended to remove “ecosystems”, as an ecosystem cannot be considered “healthy”, which we agree. However, rather than removing “ecosystem” entirely, we’ve changed the sentence to now read “… “healthy” plants or unstressed ecosystems…” to better reflect our original thought (now lines 52-53)
  - Discussion, lines 401 (and elsewhere), it was recommended to simply state “healthy” or diseased” leaves. We have decided to keep phrasing like “with no signs of foliar disease”, as we think it is important to highlight that we are specifically discussing visible signs of disease and assuming that the coffee plants in our study could have had other diseases that we did not (and would likely have been impossible to) control for.

Other changes

Introduction, line 72: In contrast to rust fungi, which are obligate biotrophs (and nearly exclusively) leaf parasites *Ophiostoma ulmi*, the causal agent of the Dutch elm disease is not an obligate biotroph. It is also not a leaf parasite, but spread by bark beetles into the wood of elm trees. This will need to be corrected. Delete citation. Potentially add another more fitting citation.

- Thank you for pointing out this discrepancy. We have removed the citation in question (Ďurkovič et al., 2013), and have adjusted the sentence as needed to reflect the reference included (Toome et al., 2010), which focused on the leaf rust *Melampsora epitea.* This change is now in lines 63-64.

Introduction, lines 76-79: literature backing this should be added here.

- Indeed, the structure of the paragraph made this unclear. The references we used to support the statement were in the previous paragraph. We have now moved this sentence to the previous paragraph and changed the wording to better clarify. It now reads “Although these preliminary findings are specific to plant genotypes and pathogen types, they indicate that…” (now lines 67-68).

Materials and methods, lines 104-133 (re: “Model species” subsection): I suggest to move this part to introduction still

- We moved this subsection to now be in the Introduction and have made some edits to ensure no repetition.

Model species subsection, line 115: But despite this extensive work on the biology of *H. vastatrix* only relatively recently surprising finding of cryptosexuality, which has a significant impact on the ability of the species to overcome resistances in the plant host and should be cited here. Carvalho, C. R., Fernandes, R. C., Carvalho, G. M., Barreto, R. W., & Evans, H. C. (2011). Cryptosexuality and the genetic diversity paradox in coffee rust, Hemileia vastatrix. *PLoS One*, *6*(11), e26387

- We now include the suggested reference (now in line 105)

Model species subsection, line 117: This is misleading. Teliospores are also ephemerally produced (which then produce basidiospores). However, despite ample attempts to infect coffee plants with basidiospores this could never been achieved suggesting that *Hemileia* is a heteroecious rust (with unknown alternate host species still to be found in its centre of origin). Thus, urediniospores are the only important spore stage for the infection of coffee plants and dispersal of the coffee rust disease. But basidispospores are also "infectious units" within the life cycle of this rust species. We just do not know which plant species they infect.

- Thank you for highlighting this misleading phrasing. We now state “…the only important infectious unit of *H. vastatrix* of coffee…” (now line 108)

Model species subsection, line 123: To my knowledge no effect of water status of leaves (or leaf cells) has been shown for rust infection.

- You are correct. We were originally referring to the water status of leaves in relation to stomatal action. However, the role of stomatal opening in the infection cycle of *H. vastatrix* is still hypothetical, and so we have decided to take out this latter part of the sentence, now stating “…suggests that greater stomatal density increases *H. vastatrix* incidence” (now lines 113-114)

Materials and methods, lines 163-165: This seems like a very coarse frame, but might serve the purpose, because it allows comparison with citation 35!? However, in general one would expect that finer grades of fruiting classes would be more appropriate.

- Indeed, this is a coarse frame, yet rather than selecting this frame to be comparable to the reference cited, we chose this coarse frame to reflect the critical threshold observed in the reference cited. To better explain this, we now provide clarification in lines 159-162, “Within each shade tree treatment, we sampled coffee plants that fell into one of two yield classes: a low fruiting class (0-200 fruiting nodes), and a high fruiting class (200+ fruiting nodes), as determined by counting fruiting nodes [41], reflecting a similar yield threshold observed by [36], where shade levels interact with CLR differently around this critical threshold.”

Materials and methods, lines 173-175: Please give the rationale for this.

- Our leaf sampling technique is based on standardized sampling protocols. We now include this clarification and the appropriate citation in lines 170-172, stating, “Within each stratum, we followed standardized leaf sampling protocols for leaf traits [8], and selected the first three recently developed, fully expanded coffee leaves from the branch tip that demonstrated signs of CLR infection for leaf trait measurements.”

Materials and methods, line 185: 10x total or 100x total magnification?

- It was 100x total magnification, and we’ve made the change, now in line 181.

Materials and methods, line 187: Please give manufacturer, city, country. Also for ImageJ at the end of the sentence.

- We now include the manufacturer information for the elemental analyzer (now line 183). For ImageJ, since no such information is available, we include the suggested reference for this free software (now reference #44).

Discussion, line 390: The biology of *Ophiostoma ulmi* and the powdery mildew of barley mentioned here are very different to rusts. While *Ophiostoma* is not an obligate biotroph, but has a close relation with the vectoring insects and growing in wood, powdery mildews are also obligate biotrophs but only infecting epidermal cells via direct penetration of the cuticula and epidermal cell wall and developing haustoria. A reference to literature on rust fungi would help.

- Indeed, given the focus of this paper on a leaf rust, references and comparisons should also be focused on leaf rust species. We’ve now changed the references here and the information included to reflect the new reference. We now state (now lines 383-386), “Indeed, changes in C assimilation rates have reflected a key tolerance mechanism in other pathogen-tolerant plant genotypes, such as *Senecio vulgari* (common groundsel) with *Coleosporium tussilaginis* [61]”, where reference #61 is https://doi.org/10.1094/PHYTO-96-0718

Conclusion, line 417: Please see my alternative text. I hope you can follow to phrase it a bit "softer". This less strong phrasing of the impact of ITV on the outcome of infection is a result of my view and knowledge of plant pathogens and rust fungi specifically. The way you end the paper (and indeed much of the paper) has a very plant centric approach. However, rusts in general and here, Hemileia vastatrix specifically, also very actively interact with the plants and have a plethora of evolutionary-ecological adaptations to do this successfully. Therefore, I suggest to keep the conclusions a bit less rigorous with respect of the plant traits governing or driving the outcome of the severity of the disease.

- We have modified the conclusion following your suggestions. It now reads (in its entirety, now lines 406-413), “Our results provide evidence that plant functional traits and their relationships shift under pathogen pressures, representing among the first lines of evidence indicating that ITV plays a role in mediating disease severity, specifically coffee leaf rust in a managed agroecosystem. In doing so, our work both advances our understanding of the functional ecology of a major crop pathogen and addresses a vital gap in our understanding of global patterns of functional trait variation. Overall, we offer first insights into the potential role of ITV in plant-pathogen-environment interactions, still only a glimpse into the response-effect co-evolution between plants and their native pathogens, highlighting the need to further explore foliar pathogens within the global functional trait space.”
